# P53 suppresses ribonucleotide reductase via inhibiting mTORC1

**DOI:** 10.18632/oncotarget.17440

**Published:** 2017-04-26

**Authors:** Zhengfu He, Xing Hu, Weijin Liu, Adrienne Dorrance, Ramiro Garzon, Peter J. Houghton, Changxian Shen

**Affiliations:** ^1^ Department of Thoracic Surgery, Sir Run Run Shaw Hospital, College of Medicine Zhejiang University, Hangzhou, Zhejiang Province, China; ^2^ College of Biology and Food Engineering, Huaihua University, Huaihua, Hunan Province, China; ^3^ Comprehensive Cancer Center, The Ohio State University, Columbus, Ohio, USA; ^4^ The Greehey Children's Cancer Research Institute, The University of Texas Health Science Center at San Antonio, San Antonio, Texas, USA

**Keywords:** mammalian target of rapamycin (mTOR), deoxyribonucleotides (dNTPs), ribonucleotide reductase (RNR), p53, nutlin-3

## Abstract

Balanced deoxyribonucleotides pools are essential for cell survival and genome stability. Ribonucleotide reductase is the rate-limiting enzyme for the production of deoxyribonucleotides. We report here that p53 suppresses ribonucleotide reductase subunit 1 (RRM1) and 2 (RRM2) via inhibiting mammalian target of rapamycin complex 1 (mTORC1). *In vitro*, cancer cell lines and mouse embryonic fibroblast cells were treated with different concentrations of pharmacological inhibitors for different times. *In vivo*, rhabdomyosarcoma Rh30 cell tumor-bearing mice were treated with rapamycin or AZD8055. Protein levels and phosphorylation status were assessed by immunoblotting and mRNA levels were determined by real time RT-PCR. Pharmacological inhibition of mTORC1 with rapamycin, mTOR kinase with AZD8055 or protein kinase B with MK2206 resulted in decrease of RRM1 and RRM2 in Rh30 cells both *in vitro* and in mouse tumor xenografts. Moreover, eukaryotic translational initiation factor 4E-binding proteins 1 and 2 double knockout mouse embryonic fibroblast cells demonstrated an elevation of RRM1 and RRM2. Furthermore, down-regulation of mTOR-protein kinase B signaling or cyclin dependent kinase 4 led to decrease of RRM1 and RRM2 mRNAs. In addition, TP53 mutant cancer cells had elevation of RRM1 and RRM2, which was reduced by rapamycin. Importantly, human double minute 2 inhibitor nutlin-3 decreased RRM1 and RRM2 in TP53 wild type rhabdomyosarcoma Rh18 but not in TP53 mutated Rh30 cells. Our data demonstrated that mTOR enhances the cap-dependent protein translation and gene transcription of RRM1 and RRM2. Our findings might provide an additional mechanism by which p53 maintains genome stability.

## INTRODUCTION

Increased or imbalanced deoxyribonucleotides (dNTPs) lead to genome instability, a hallmark of cancer cells [1], while decreased dNTP level impairs cell survival [2–4]. Ribonucleotide reductase (RNR) catalyzes the rate-limiting step in the production of dNTPs from ribonucleotides, and its expression and activity are tightly controlled in all organisms under normal growth and stressful conditions [2–4]. Yeast RNR is composed of either two identical large subunits of Rnr1, Rnr3, or Rnr1/Rnr3, and two small subunits Rnr2 and Rnr4. In yeast, the activity of RNR is mainly regulated by allosteric feedback control, i.e., high levels of dNTPs inhibit the enzyme activity of RNR and consequently restore dNTPs to physiological concentrations [4–5]. The mammalian RNR is composed of two identical ribonucleotide reductase large subunit 1 (RRM1) and two small subunits of either RRM2 or p53R2. p53R2 is DNA damage inducible and is regulated by p53. Both RRM1 and RRM2 are dynamically regulated during the cell cycle progression [2–5]. The activity of RNR is principally controlled by the level of RRM2 in mammalian cells [6]. Balanced dNTPs pools are essential for the accurate and efficient DNA synthesis for replication and repair, defects of which lead to cell death, genome instability, or anti-cancer drug resistance [3–5]. Rapidly proliferating tumor cells encounter frequent metabolic stress due to the transient and long-term lack of nutrients, oxygen, and growth factors [1, 7, 8]; however, the mechanisms by which cancer cells maintain the activity of RNR, and thereby the level of dNTPs under ever-changing microenvironment is not fully understood.

In response to DNA damage, there is a 6-8 fold increase of intracellular dNTPs in yeast through several mechanisms [9], including upregulation of the transcription of the subunits of RNR genes [10], degradation of the RNR holyenzyme inhibitor Sml1 [11], and cytoplasmic translocation of Rnr2 and Rnr4 from nucleus [12, 13]. These increased protein levels, released enzyme activity and subcellular positioning of RNR holyenzyme result in rapid increase of intracellular dNTPs. The increase of dNTPs in response to DNA damage is essential for yeast cells to survive DNA damage via translesion DNA synthesis because incompletely replicated DNA results in cell death [9, 14]. A previous study showed that the target of rapamycin (TOR) sustains Rad53-mediated induction of Rnr1 and Rnr3, and promotes cell survival but at the cost of increased mutation rate in response to DNA damage in yeast [15]. TOR kinase is a conserved member of the PI3K-related kinase family. Mammalian target of rapamycin (mTOR) forms functionally distinct complexes, mTOR complex 1 (mTORC1) and 2 (mTORC2). mTORC1 is rapamycin sensitive and controls cell growth and proliferation by enhancing protein synthesis through the eukaryotic translational initiation factor 4E (eIF-4E) and S6 kinase 1 (S6K1) signaling pathways. mTORC2 stimulates the activation of protein kinase B (PKB/AKT) by phosphorylating AKT at serine 473. In addition, there is an AKT-mTORC1-S6K1-IRS1/2 negative feedback signaling. mTORC1 acts as an integrator of multiple intracellular and extracellular signals, and regulates cell growth, proliferation, differentiation, metabolism, and survival [16–19]. Deregulation of AKT-mTOR signaling has been found in most cancers and the mTOR axis is a common target for the development of molecular targeted therapies for cancer [18, 20], however, whether RNR is regulated by mTOR in mammalian cells and deregulation of RNR contributes to the carcinogenesis and anti-cancer drug resistance by mTOR signaling is unknown.

In this study, to explore the potential role for mTOR signaling in the regulation of RNR in cancer cells, we have determined the mRNA and protein levels of *RRM1* and *RRM2* in cell culture and mouse tumor xenografts following pharmacological and genetic inhibition of mTOR signaling. Our results demonstrated that the mTOR pathway positively controls both the gene transcription and protein translation of *RRM1* and *RRM2*, and p53 suppresses RRM1 and RRM2 via inhibition of mTORC1.

## RESULTS

### mTORC1 enhances the cap-dependent protein translation of RRM1 and RRM2

**Figure 1:**
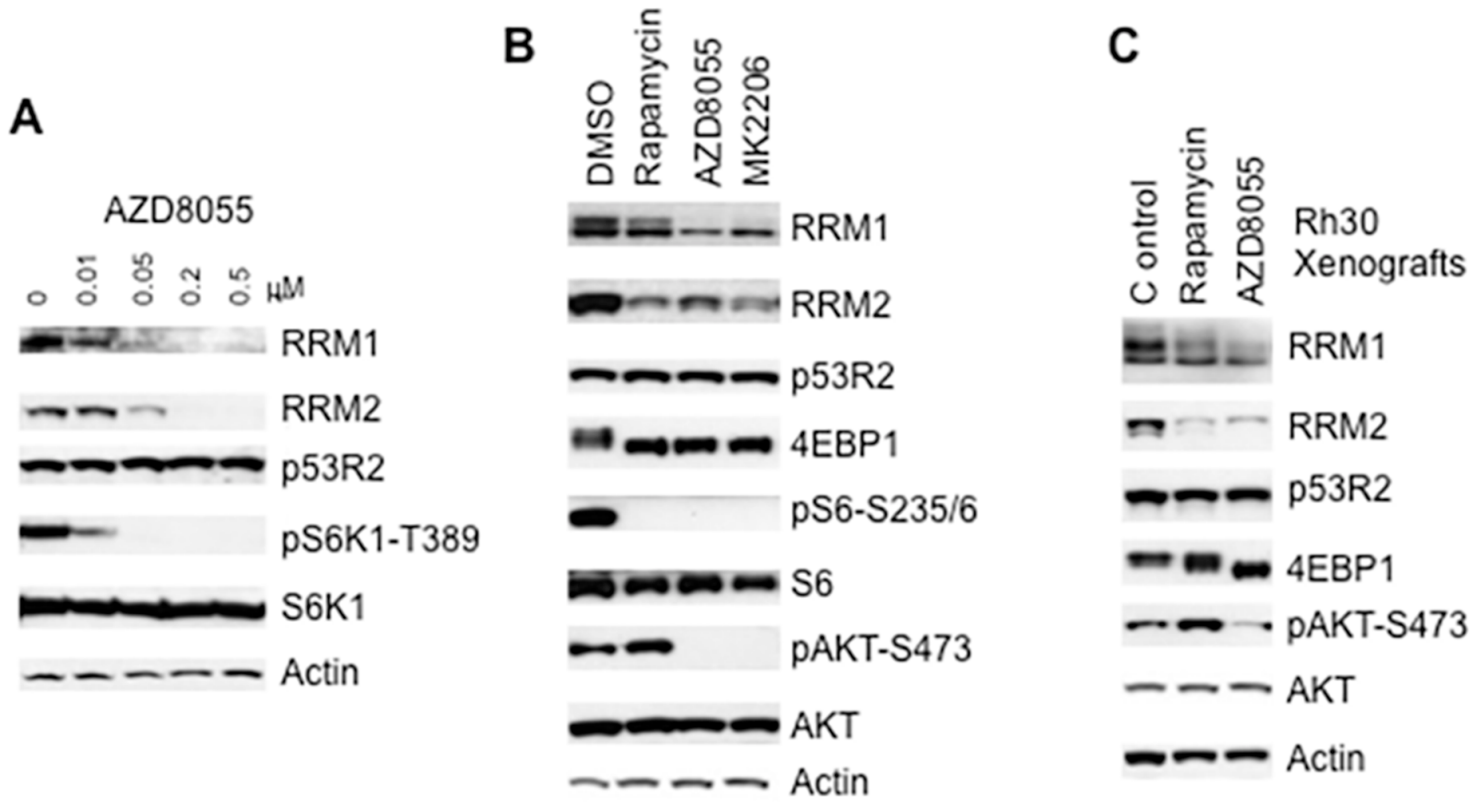
Inhibition of mTOR signaling results in decrease of RRM1 and RRM2 **(A)** Rh30 cells were treated with AZD8055 at the concentrations as indicated for 24 hr. Total proteins were extracted for immunoblotting of RRM1, RRM2, p53R2, pS6K1-T389 and S6K1. **(B)** Rh30 cells were treated with rapamycin (100 ng/mL), AZD8055 (1 μM), or MK2206 (10 μM) for 24 hr. Total proteins were extracted for immunoblotting of RRM1, RRM2, p53R2, 4E-BP1, pS6-S235/6, AKT and pAKT-S473. **(C)** Pediatric rhabdomyosarcoma Rh30 tumor xenograft models were propagated subcutaneously in SCID mice and were treated with mTOR kinase inhibitor AZD8055 at 20 mg/kg/day or rapamycin at 5 mg/kg per day. Tumors were harvested 24 hr post treatment on day 1. Total proteins were extracted for immunoblotting. Actin served as loading controls.

**Figure 2:**
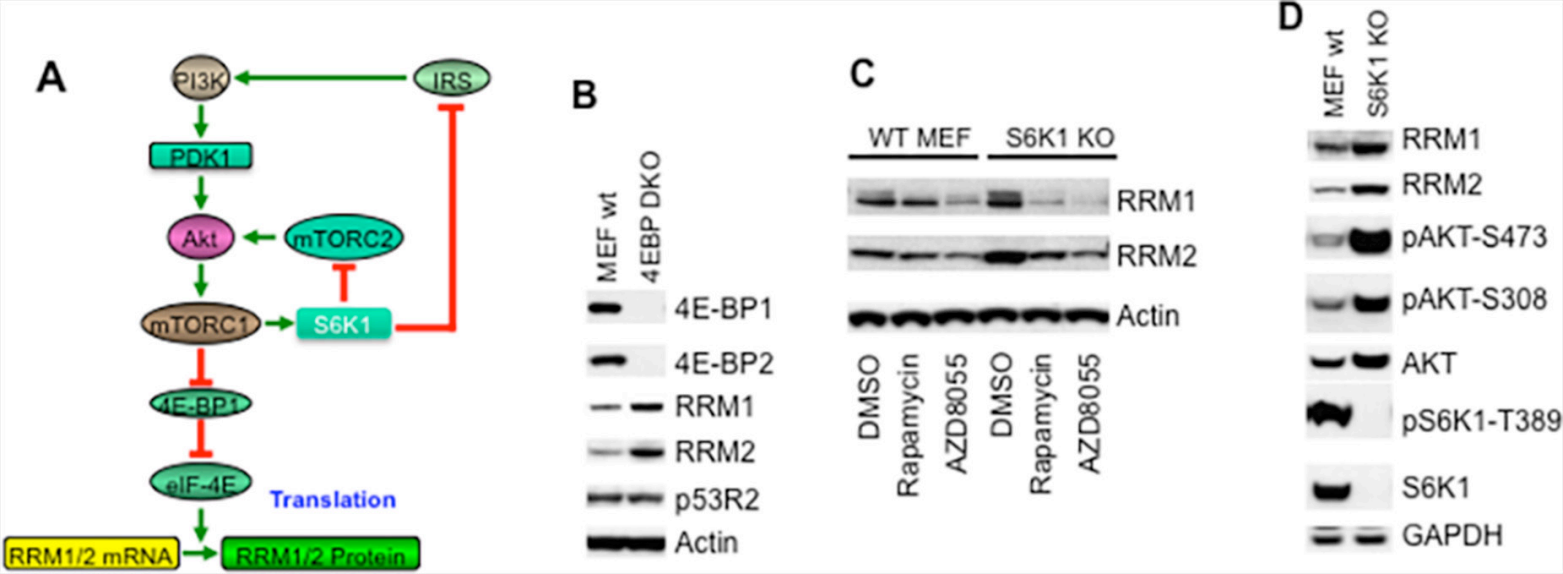
mTOR signaling increases RRM1 and RRM2 via cap-dependent protein translation **(A)** Scheme of the regulation of the cap-dependent protein translation of RRM1 and RRM2 through the PI3K/AKT/mTORC1/4E-BP1 signaling pathway. **(B)** Total proteins of wild type (MEF WT) and *4E-BP1/2* double knockout (4EBP DKO) MEF cells were extracted to detect 4E-BP1, 4E-BP2, RRM1, RRM2 and p53R2 by immunoblotting. **(C)** Wild type (WT MEF) and *p70S6K1* knockout (S6K1 KO) MEF cells were treated with rapamycin (100 ng/mL) or AZD8055 (1 μM) for 24 hr. Total proteins were extracted for immunoblotting of RRM1 and RRM2. **(D)** Total proteins of wild type (WT MEF) and *p70S6K1* knockout (S6K1 KO) MEF cells were extracted to detect S6K1, pS6K1-T389, pAKT-S473, pAKT-T308, AKT, RRM1 and RRM2 by immunoblotting. GAPDH and Actin served as loading controls.

The mTOR kinase specific inhibitor AZD8055 potently inhibits both mTORC1 and mTORC2 [21–23]. To determine the regulation of RNR by mTOR signaling, we treated Rh30 cells with different concentrations of AZD8055 and assessed the three mammalian RNR subunits, RRM1, RRM2 and p53R2 by immunoblotting. AZD8055 at 10 nM decreased pS6K1-T389 signal, indicating suppression of mTORC1. AZD8055 reduced the protein levels of both RRM1 and RRM2 at 50 nM, while p53R2 was not affected by AZD8055 even at 500 nM for 24 hr (Figure 1A). To test whether the reduction of RRM1 and RRM2 results from the down-regulation of cap-dependent translation following inhibition of mTORC1 pathway by AZD8055, we used rapamycin, AZD8055, and MK2206 to inhibit mTORC1, mTOR kinase, and AKT kinase [24], respectively. Either AZD8055 or MK2206 treatment resulted in decrease of both RRM1 and RRM2 but not p53R2, accompanied with the disappearance of both pS6-S235/6 and pAKT-S473 signals, and dephosphorylation of 4E-BP1 (Figure 1B). Rapamycin treatment led to dephosphorylation of 4E-BP1 and disappearance of pS6-S235/6 signal, indication of mTORC1 signaling inhibition. Moreover, rapamycin increased the pAKT-S473 signal while potently reduced RRM1 and RRM2 (Figure 1B). Similar results were observed in Rh30 mouse tumor xenografts treated with either rapamycin or AZD8055 (Figure 1C). As mTORC1 is a downstream effector of the mTORC2-AKT signaling pathway [19], our data clearly indicate that pharmacological inhibition of mTORC1 is sufficient to reduce RRM1 and RRM2 regardless of the its negative feedback activation of the upstream AKT signaling, and suggest that mTORC1/eIF-4E cap-dependent protein translation may be required to maintain the protein levels of RRM1 and RRM2 (Figure 2A). To further support this conclusion, we assessed RRM1 and RRM2 in *4E-BP1* and *4E-BP2* double knockout MEF cells (4E-BP KO). In comparison to wild type MEFs, *4E-BP* DKO MEFs demonstrated elevated RRM1 and RRM2, but not p53R2 (Figure 2B). Taken together, these data suggest that mTORC1/eIF-4E cap-dependent protein translation plays an important role in the control of both RRM1 and RRM2 (Figure 2A).

### S6K1 signaling may suppress RRM1 and RRM2 via the negative feedback inhibition of AKT circuit

Another established downstream target of mTORC1 is S6K1 [19]. To assess whether S6K1 signaling plays any role for the regulation of RNR by mTOR, we treated wild type and *S6K1* knockout MEF cells (*S6K1* KO MEFs) with rapamycin or AZD8055 and checked RRM1 and RRM2 by immunoblotting. Unexpectedly, depletion of S6K1 resulted in apparent up-regulation of both RRM1 and RRM2; however either rapamycin or AZD8055 still decreased RRM1 and RRM2 in both wild type and *S6K1* KO MEFs (Figure 2C). To test whether the up-regulation of RRM1 and RRM2 in *S6K1* KO MEFs results from activation of mTORC2 and AKT signaling following depletion of S6K1 (Figure 2A), we assessed the activity of AKT signaling in these MEFs. In *S6K1* KO MEFs, there were no detectable S6K1 and pS6K1-T389 signals, indication of *S6K1* knockout. Compared to wild type MEFs, *S6K1* KO MEFs demonstrated enhanced pAKT-S473 and pAKT-S308 signals, indicating activation of AKT signaling. As expected, there was increased RRM1 and RRM2 in *S6K1* KO MEFs when compared with that of wild type MEFs (Figure 2D). Since S6K1 inhibits AKT signaling via suppressing IRS1 and mTORC2 [16], our results suggest that S6K1 may suppress RRM1 and RRM2 via the negative feedback inhibition of AKT signaling.

### The mTOR pathway promotes the gene transcription of *RRM1* and *RRM2*

**Figure 3:**
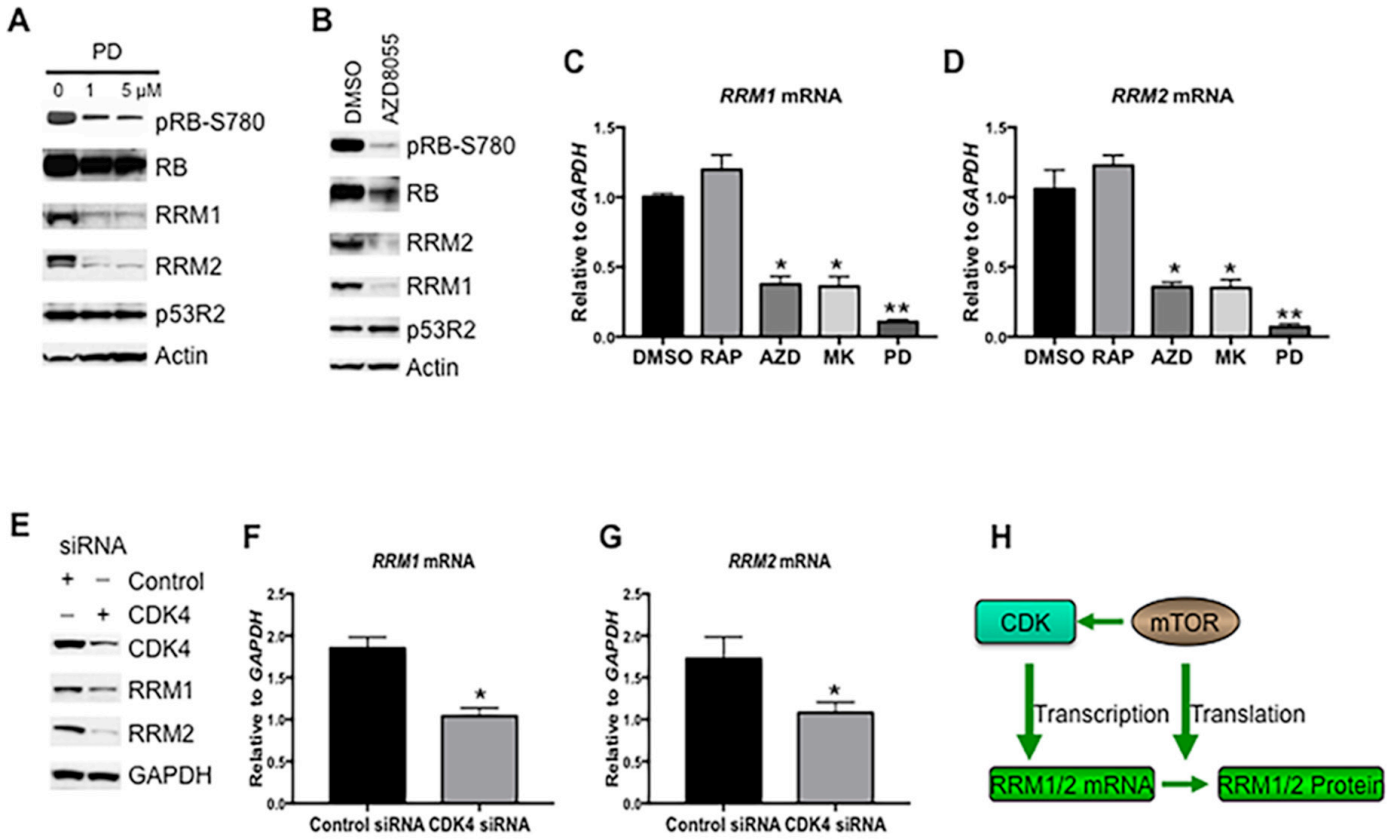
RRM1 and RRM2 are transcriptionally enhanced by mTOR and CDK4/6 **(A)** Rh30 cells were treated with PD0332991 (PD) at the concentrations as indicated for 24 hr. Total proteins were extracted for immunoblotting of RRM1, RRM2, p53R2, pRB-S780 and RB. **(B)** Rh30 cells were treated with 1 μM AZD8055 for 24 hr. Total proteins were extracted for immunoblotting of RRM1, RRM2, p53R2, pRB-S780 and RB. **(C)** Rh30 cells were treated with rapamycin (100 ng/mL), AZD8055 (1 μM), MK2206 (10 μM), or PD0332991 (5 μM) for 24 hr. Total RNA was extracted to detect *RRM1* mRNA by real-time RT-PCR with *GAPDH* as internal control. Relative quantity of *RRM1* mRNA was plotted. *P<0.05; **P<0.01 vs DMSO. **(D)** Total RNA from **(C)** was used to detect *RRM2* mRNA by real-time RT-PCR with *GAPDH* as internal control. Relative quantity of *RRM2* mRNA was plotted. *P<0.05; **P<0.01 vs DMSO. **(E)** Rh30 cells were transfected with siRNAs of control, or CDK4. 72 hr later, total proteins were extracted for immunoblotting. **(F)** Rh30 cells were treated as in **(E)**, total RNA was extracted to detect *RRM1* mRNAs by real-time RT-PCR with *GAPDH* as internal control. Relative quantity of mRNA was plotted. *P<0.05 vs DMSO. **(G)** Rh30 cells were treated as in **(E)**, total RNA was extracted to detect *RRM2* mRNAs by real-time RT-PCR with *GAPDH* as internal control. Relative quantity of mRNA was plotted. *P<0.05 vs DMSO. **(H)** Simplified model of the regulation of RRM1 and RRM2 by mTOR signaling. RAP: rapamycin; MK: MK2206; AZD: AZD8055; PD: PD0332991.

It has been shown that the gene transcription of *RRM1* and *RRM2* is regulated during the cell cycle and controlled by cyclin D dependent kinase (CDK4/6) [25, 26]. To assess whether the mTOR pathway regulates the gene transcription of *RRM1* and *RRM2* via promoting CDK4/6 activity, we first treated Rh30 cells with different concentrations of CDK4/6 specific inhibitor PD0332991 [27, 28] and determined RRM1 and RRM2 protein levels by immunoblotting. As shown in Figure 3A, pharmacological inhibition of CDK4/6 by PD0332991 resulted in dephosphorylation of RB at serine 780, a marker of inhibition of the activity of CDK4/6, and a decrease of both RRM1 and RRM2 but not p53R2 (Figure 3A), indicating RRM1 and RRM2 is regulated by CDK4/6. Next, we treated Rh30 cells with AZD8055 and assessed pRB-S780, RRM1 and RRM2 protein levels by immunoblotting. AZD8055 decreased the phosphorylation of RB at serine 780, which was accompanied with a reduction of RRM1 and RRM2 (Figure 3B). Depletion of S6K1 increased the activity of mTORC2-AKT (Figure 2D), which may in turn promote the activity of CDK4/6 and hence the gene transcription of *RRM1* and *RRM2*. To test this hypothesis, we treated Rh30 cells with rapamycin, AZD8055, MK2206, or PD0332991 for 12 hr and checked the mRNA levels of *RRM1* and *RRM2* by real time PCR. PD0332991 significantly downregulated the mRNA levels of both *RRM1* (Figure 3C, P<0.01) and *RRM2* (Figure 3D, P<0.01). Though rapamycin reduced the protein levels of RRM1 and RRM2 (Figure 1B and 1C), it did not significantly change the mRNA levels of either *RRM1* or *RRM2*, indicating the importance of mTORC1/4E-BP1 in the maintenance of the protein levels of RRM1 and RRM2 via promoting protein translation. In contrast, both AZD8055 and MK2206 significantly decreased the mRNA levels of *RRM1* and *RRM2* (all P<0.05). We further downregulated CDK4 by siRNA in Rh30 cells and checked the protein and mRNA levels of *RRM1* and *RRM2* by immunoblotting and real time PCR, respectively. Consistent with the pharmacological results, down-regulation of CDK4 by siRNA led to decrease of both the protein (Figure 3E) and mRNA levels of *RRM1* (Figure 3F, P<0.05) and *RRM2* (Figure 3G, P<0.05). Taken together, our results indicate that mTOR increases the gene transcription via CDKs as well as mTORC1/eIF-4E cap-dependent translation of *RRM1* and *RRM2* (Figure 3H), suggesting the critical importance of mTOR in the control of RNR.

### p53 regulates RRM1 and RRM2 via the mTORC1 pathway

**Figure 4:**
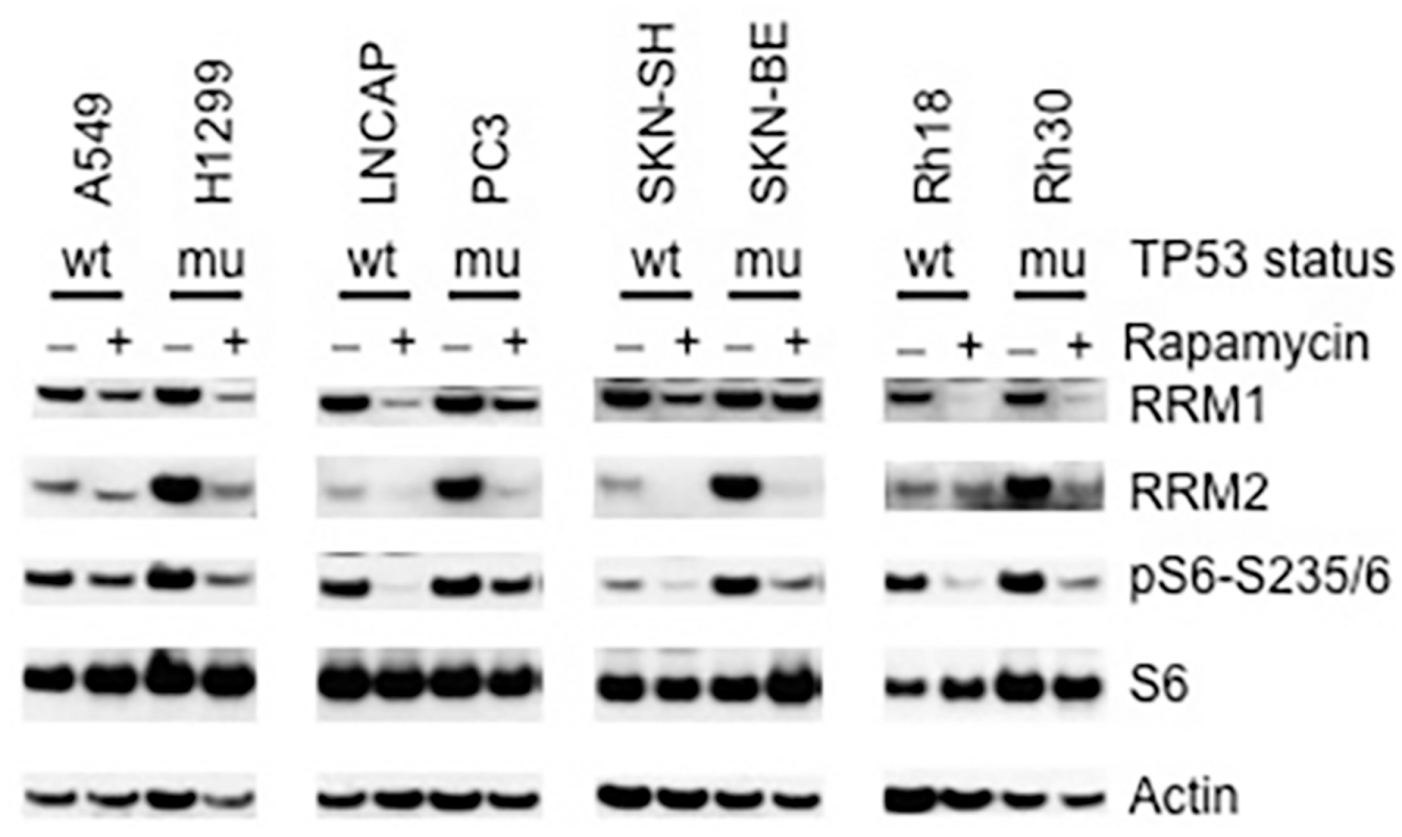
p53 suppresses RRM1 and RRM2 TP53 wild type (A549, LNCAP, SKN-SH and Rh18) and mutant (H1299, PC3, SKN-BE and Rh30) cells were treated with rapamycin (100 ng/mL) for 24 hr. Total proteins were extracted for immunoblotting of RRM1, RRM2, S6, and pS6-235/6. wt: TP53 wild type; mu: TP53 mutated.

It is well known that p53 inhibits the functions of mTORC1 via multiple mechanisms [29], while most cancer cells have lost functional p53 circuit and acquired enhanced AKT-mTOR signaling [20, 30, 31]. To explore whether loss-function of p53 affects RNR in cancer cells, we compared the RRM1 and RRM2 levels of TP53 wild type with that of TP53 mutant cancer cell lines, including lung carcinoma A549 (TP53 wild type) and H1299 (TP53 mutant), pancreatic cancer LNCAP (TP53 wild type) and PC3 (TP53 mutant), neuroblastoma SKN-SH (TP53 wild type) and SKN-BE (TP53 mutant), rhabdomyosarcoma Rh18 (TP53 wild type) and Rh30 (TP53 mutant) cells. In all the four types of tumors, TP53 mutant cancer cells had relatively enhanced phosphorylation of S6 in comparison to those of TP53 wild type cancer cells, an indication of increased activity of mTORC1 in the cancer cells with loss-function of p53. There was slight increase of RRM1 in TP53 mutant cancer cells. However, very impressively, compared with that of TP53 wild type cancer cells, RRM2 was dramatically upregulated in all TP53 mutant cancer cells (Figure 4). Consistent with regulation of RRM1 and RRM2 by mTORC1, rapamycin treatment led to downregulation of both RRM1 and RRM2 in these TP53 wild type and mutant cancer cells, accompanied with reduction of pS6-S235/6. These results indicate the upregulation of RNR subunits especially RRM2 in TP53 mutant cancer cells is at least partially due to the loss of the negative control of mTORC1 by p53.

### Restoration of p53 function by an HDM2 inhibitor leads to decrease of RRM1 and RRM2 in TP53 wild type cancer cells

**Figure 5:**
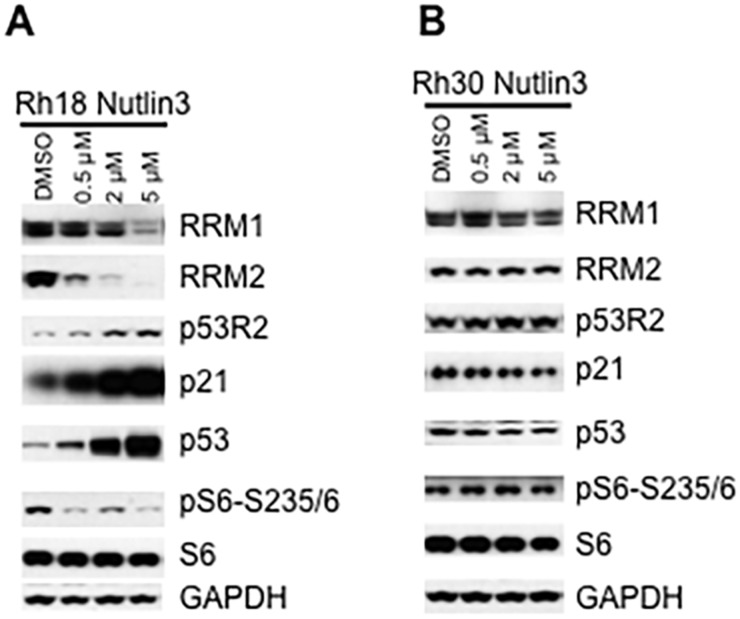
Inhibition of HDM2 by nutlin-3 decreases RRM1 and RRM2 in cancer cells with wild type TP53 **(A)** Rh18 cells were treated with different concentrations of nutlin-3 as indicated for 24 hr. Total proteins were extracted for immunoblotting of RRM1, RRM2, γH2AX, p21, p53R2, p53, S6, pS6-235/6 and 4E-BP1. **(B)** Rh30 cells were treated with different concentrations of nutlin-3 as indicated for 24 hr. Total proteins were extracted for immunoblotting of RRM1, RRM2, γH2AX, p21, p53R2, p53, S6, pS6-235/6 and 4E-BP1. GAPDH and Actin served as loading controls.

Our above observation demonstrates that RNR subunits, especially RRM2 are under control of p53. One of the mechanisms of loss-function of p53 is the enhanced activity of p53 ubiquitin ligase E3, human double minute 2 (HDM2) [31]. Our data suggest that reactivation of p53 in cancer cells with wild type TP53 might suppress mTORC1-RRM1/2. To test this hypothesis, we used the HDM2-amplified Rh18 cell line as a model to reactivate the function of p53 via inhibiting HDM2 by nutlin-3 [32]. As shown in Figure 5A, nutlin-3 significantly increased p53 protein levels as well as the p53 functions as evidenced by the induction of p53R2 and p21, transcriptional targets of p53, while decreased RRM2 and RRM1. In agreement with the functions of p53 in suppressing mTORC1, nutlin-3 suppressed the phosphorylation of S6 with the increase of nutlin-3 dosage. In sharp contrast, nutlin-3 did not alter the protein levels of p53, p53R2, p21, RRM1 and RRM2, and the phosphorylation of S6 in TP53 mutated Rh30 cells (Figure 5B). These results demonstrated that inhibition of HDM2 efficiently leads to decrease of RRM1 and RRM2 via p53-mTORC1 signaling in TP53 wild type Rh18 but not TP53 mutant Rh30 cells.

## DISCUSSION

In the present study, we have found that mTOR positively regulates the gene transcription and cap-dependent protein translation of both *RRM1* and *RRM2* in mammalian cells (Figure 3H). In addition, reactivation of p53 with HDM2 inhibitor nutlin-3 reduced RRM1 and RRM2.

For survival, cells exploit a set of translesion DNA polymerases to bypass DNA damage to finish DNA replication under conditions of DNA damages. These translesion DNA polymerases need much higher dNTPs levels than normal DNA polymerases. The consequence of this translesion DNA synthesis is the increased mutation rate thereby contributing to DNA damage-induced gene mutation [14, 33]. It has been estimated that tumor cells have concentrations of 6-11 fold of the four dNTPs over normal cells, which may contribute to the enhanced proliferation and mutation rate of cancer cells [34]. However, the pathophysiology and mechanisms of the deregulation of dNPTs level in tumorigenesis and anti-cancer drug resistance remains to be determined. The activity of mammalian RNR is mainly controlled by the level of RRM2 and up-regulation of RRM2 in mice leads to lung cancer [2, 5]. Consequently, RRM2 has been proposed as biomarker for the prognoses of some cancer patients [35, 36]. In yeast, rapamycin inhibition of TORC1 reduced the DNA damage-mediated mutations through down-regulation of RNR [15]. In the present study, we showed that mTOR controlled the mRNA and protein levels of both *RRM1* and *RRM2* in mammalian cells. mTOR lies at the hub of intracellular and extracellular signal transduction pathways and the mTOR kinase-mediated signaling is deregulated in most cancers [16–20]. Our findings suggest that regulation of RNR might contribute to the promotion of tumorigenesis by mTOR and ‘addiction’ of cancer cells on mTOR signaling.

Stressful conditions, such as growth factor deprivation, starvation, hypoxia and some DNA damaging agents, inhibit mTORC1 signaling through multiple mechanisms [7, 37, 38]. It has been well demonstrated that p53 suppresses mTORC1 signaling, leading to the proposal that one of the mechanisms by which p53 suppresses tumorigenesis is to downregulate mTORC1 [29, 37], but the underlying molecular mechanisms remain to be determined. Our results indicate that RRM2 as well as RRM1 are positively controlled by AKT-mTOR signaling while being suppressed by p53. Given that increased activity of RNR and hence dNTPs promote tumorigenesis, cell proliferation and survival [2–6], our findings suggest mutation of *TP53* may result in deregulation of mTORC1 and concomitant upregulation of RNR to render cancer cells to survive stressful conditions but at the cost of increased mutation rate.

One of the mechanisms of loss-function of p53 is the enhanced activity of p53 ubiquitin ligase E3, HDM2 [31]. Recent advances have shown that targeting HDM2 to reactivate p53 is a very promising strategy for the development of cancer therapeutics for TP53 wild type cancers. Currently, there are numerous HDM2 inhibitors in both preclinical and clinical trials, with nutlin family the most successful model [39, 40]. We showed that reactivation of p53 by nutlin-3 mediated inhibition of HDM2 reduced RRM1 and RRM2 in TP53 wild type but not TP53 mutated cancer cells. Regarding that the activity of RNR is essential for the survival of rapidly proliferating cancer cells, our findings suggest that downregulation of RNR may contribute to the pharmacology of HDM2 inhibitors.

It has been reported that the gene transcription of *RRM1* and *RRM2* is regulated by cyclin dependent kinases (CDKs) during G1 and S phases of the cell cycle [4–6]. In agreement with the finding that mTOR signaling promotes G1 and S phase progression by enhancing the activity of CDKs [16–19], we found that mTOR increased the gene transcription of *RRM1* and *RRM2*. Nevertheless, the underlying molecular mechanisms remain to be determined. It has been shown that E2F4 binds to the *RRM2* promoter, and E2F4 and p130/p107 are part of the 'DREAM' complex [41], which binds to E2F sites [42]. In addition, most recent meta-analysis has shown that DREAM proteins bind to *RRM1* and *RRM2* promoters, and *RRM1* and *RRM2* mRNA levels decrease after p53 activation [43]. Whether mTOR regulates *RRM1* and *RRM2* transcription through E2F4/p130/p107 as part of DREAM warrants further investigation.

In summary, our results demonstrated that both RRM1 and RRM2 were positively controlled by mTOR signaling while suppressed by p53 signaling. Our study may have discovered a novel mechanism by which mTOR and p53 signaling pathways regulate the initiation and progression of tumorigenesis, and have provided new strategies for the development of anti-cancer drugs.

## MATERIALS AND METHODS

### Drugs

Rapamycin was from the NCI drug repository. AZD8055, MK2206, PD0332991 and Nutlin 3 were from Selleck Chemicals (Houston, TX). The detailed information of these drugs is listed in Supplementary Table 1.

### Cells and siRNA

Rhabdomyosarcoma Rh30 and Rh18, lung carcinoma A549 and H1299, pancreatic cancer LNCAP and PC3, neuroblastoma SKN-SH and SKN-BE cells were from American Type Culture Collection (ATCC, Rockville, MD). A549, H1299, LNCAP, PC3, SKN-SH and SKN-BE cells were cultured in DMEM (GIBCO) supplemented with 10% heat-inactivated FBS (GIBCO). Rh18 and Rh30 cells were cultured in RMPI 1640 (GIBCO) supplemented with 10% heat-inactivated FBS. 4E-BP1/2 double knock-out MEFs were from Dr. Nahum Sonenberg (McGill University, Canada), and S6K1 knock-out MEFs were provided by Dr. George Thomas (University of Cincinnati). MEF cells were cultured in DMEM supplemented with 10% heat-inactivated FBS. Control and ON-TARGETplusSMARTpool siRNAs of CDK4 were purchased from Dharmacon (Chicago, IL). Lipofectamine 2000 was from Invitrogen (Carlsbad, CA) and transfection of siRNA in cells was performed according to the manufacture's instructions.

### Immunoblotting

Cells were lysed on ice in RIPA lysis buffer (Cell Signaling Technology, Boston, MA) supplemented with protease inhibitors and phosphatase inhibitor (Roche), and 1mM PMSF (Sigma). Equal amount of protein (20 μg) was separated on sodium dodecyl sulfate polyacrylamide gels and transferred onto polyvinylidene fluoride membranes (Millipore). After being blocked with 5% non-fat dry milk in Tris-buffered saline and Tween 20 (10 mM Tris-HCl, pH 8.0, 100 mM NaCl and 0.05% Tween 20, TBST) at room temperature for 1 h, the membranes were incubated overnight at 4°C with primary antibodies, followed by incubation with horseradish peroxidase-conjugated secondary antibodies. After extensive washing with TBST, protein bands were visualized by an ECL plus chemiluminescence kit (Pierce). The following are the used antibodies: S6, pS6 (S235/236), AKT, pAKT (S473), pAKT (S308), S6K1, pS6K1 (T89), 4E-BP1, 4E-BP2, p21, p53, CDK4, β-Actin, GAPDH, Rb, pRb (S780), RRM1 (All from Cell Signaling Technology); RRM2 (Sigma), p53R2 (Abcam).

### RNA isolation, cDNA synthesis and RT-PCR

Total RNA from cultured cells was extracted with mirVana miRNA Isolation Kit (Ambion) according to the total RNA isolation protocol. Reverse transcription were performed using the High Capacity RNA-to-cDNA kit according to the manufacturer's instructions. The relative levels of target gene mRNA to control *GAPDH* were determined by quantitative real-time PCR (qRT-PCR) in 7900HT Fast Real-Time PCR System using the TaqMan® Universal Mastermix II. The PCR program consisted of an initial denaturation cycle (5 min at 95°C) followed by 40 cycles of denaturation (15 sec at 95°C) and annealing and elongation (60 sec at 60°C). A melting curve analysis was added at the end of the program. Human *RRM1* and *RRM2* expression was quantified in real-time with *RRM1* and *RRM2* specific FAM dye-labeled MGB-probes and normalized to *GAPDH* (Applied Biosystems). The data were analyzed by the 2^-ΔΔCt^.

### Solid tumor xenografts studies

CB17SC scid^-/-^ female mice were purchased from Taconic Farms (Germantown, NY) and used to propagate subcutaneously implanted tumors as previously described [21, 22]. All mice were maintained under barrier conditions and experiments were conducted using protocols and conditions approved by the animal care and use committee of The Ohio State University (IACUC protocol 2010A00000192). This study was approved by the ethic committee of The Ohio State University. All mice were maintained on a 12 h light/dark cycle in a temperature room at 22-25°C and allowed free access to food and water. AZD8055 was administered P.O. daily at 20 mg/kg per day. Rapamycin was dissolved in DMSO (5% final concentration) and diluted in 5% Tween-80 in water and administered I.P. daily at a dose of 5 mg/kg. Tumors were harvested post treatment on day 4. Snap-frozen samples were lysed with RIPA buffer and analyzed by immunoblotting.

### Statistical analyses

Graphs were constructed using GraphPad Prism (Graphpad Software, San Diego, CA). All data are presented as mean ± SEM. Statistical significance was determined by unpaired two-tailed *t* tests or two-way ANOVAs. *P* < 0.05 was considered statistically significant.

## SUPPLEMENTARY MATERIALS TABLES


