# Supplementary material for: P53 suppresses ribonucleotide reductase via inhibiting mTORC1
Source: Oncotarget. 2017 Apr 26;8(25):41422–31. doi: 10.18632/oncotarget.17440 (PMC5522210; doi:10.18632/oncotarget.17440)
Supplement: Supplementary file 1 [file oncotarget-08-41422-s001.pdf]

P53 suppresses ribonucleotide reductase via inhibiting mTORC1

Supplementary Materials

SUPPLEMENTARY TABLE

Supplementary Table 1: Chemical inhibitors used in this study

Name	Target(s)	Provider	References
AZD8055	mTORC1, mTORC2	Selleck Chemicals	1–3
MK2206	AKT	Selleck Chemicals	4
Rapamycin	mTORC1	NCI drug repository	5, 6
PD0332991	CDK4/6	Selleck Chemicals	7, 8
Nutlin 3	HDM2	Selleck Chemicals	9–11

REFERENCES

1. Chresta CM, Davies BR, Hickson I, Harding T, Cosulich S, Critchlow SE, Vincent JP, Ellston R, Jones D, Sini P, James D, Howard Z, Dudley P, et al. AZD8055 is a potent, selective, and orally bioavailable ATP-competitive mammalian target of rapamycin kinase inhibitor with *in vitro* and *in vivo* antitumor activity. Cancer Res. 2010; 70:288-98.
2. Houghton PJ, Gorlick R, Kolb EA, Lock R, Carol H, Morton CL, Keir ST, Reynolds CP, Kang MH, Phelps D, Maris JM, Billups C, Smith MA. Initial testing (stage 1) of the mTOR kinase inhibitor AZD8055 by the pediatric preclinical testing program. Pediatr Blood Cancer. 2012:58:191-9.
3. Shen C, Houghton PJ. The mTOR pathway negatively controls ATM by up-regulating miRNAs. Proc Natl Acad Sci U S A. 2013; 110:11869-74.
4. Gorlick R, Maris JM, Houghton PJ, Lock R, Carol H, Kurmasheva RT, Kolb EA, Keir ST, Reynolds CP, Kang MH, Billups CA, Smith MA. Testing of the Akt/PKB inhibitor MK-2206 by the pediatric preclinical testing program. Pediatr Blood Cancer. 2012; 59:518-24.
5. Sengupta S, Peterson TR, Sabatini DM. Regulation of the mTOR complex 1 pathway by nutrients, growth factors, and stress. Mol Cell. 2010; 40:310-22.
6. Wullschleger S, Loewith R, Hall MN. TOR signaling in growth and metabolism. Cell. 2006; 124:471-84.
7. Fry DW, Harvey PJ, Keller PR, Elliott WL, Meade M, Trachet E, Albassam M, Zheng X, Leopold WR, Pryer NK, Toogood PL. Specific inhibition of cyclin-dependent kinase 4/6 by PD 0332991 and associated antitumor activity in human tumor xenografts. Mol Cancer Ther. 2004; 3:1427-38.
8. Saab R, Bills JL, Miceli AP, Anderson CM, Khoury JD, Fry DW, Navid F, Houghton PJ, Skapek SX. Pharmacologic inhibition of cyclin-dependent kinase 4/6 activity arrests proliferation in myoblasts and rhabdomyosarcoma-derived cells. Mol Cancer Ther. 2006; 5:1299-308.
9. Patton JT, Mayo LD, Singhi AD, Gudkov AV, Stark GR, Jackson MW. Levels of HdmX expression dictate the sensitivity of normal and transformed cells to Nutlin-3. Cancer Res. 2006; 66:3169-76.
10. Feng FY, Zhang Y, Kothari V, Evans JR, Jackson WC, Chen W, Johnson SB, Luczak C, Wang S, Hamstra DA. MDM2 inhibition sensitizes prostate cancer cells to androgen ablation and radiotherapy in a p53-dependent manner. Neoplasia. 2016; 18:213-22.
11. Deben C, Wouters A, Op de Beeck K, van Den Bossche J, Jacobs J, Zwaenepoel K, Peeters M, Van Meerbeeck J, Lardon F, Rolfo C, Deschoolmeester V, Pauwels P. The MDM2-inhibitor Nutlin-3 synergizes with cisplatin to induce p53 dependent tumor cell apoptosis in non-small cell lung cancer. Oncotarget. 2015; 6:22666-79. doi: 10.18632/oncotarget.4433.
